# Treatment of unicameral bone cyst: systematic review and meta analysis

**DOI:** 10.1007/s11832-014-0566-3

**Published:** 2014-02-26

**Authors:** Muayad Kadhim, Mihir Thacker, Amjed Kadhim, Laurens Holmes

**Affiliations:** 1 Department of Orthopaedic Surgery, Alfred I. DuPont Hospital for Children, 1600 Rockland Road, Wilmington, DE 19803 USA; 2 Sawaf Institute for Medical Imaging, Victoria Bridge, Damascus Syria

**Keywords:** Unicameral Bone Cyst, Success and failure rate, Treatment modalities, Healing, Persistence and recurrence

## Abstract

**Purpose:**

Different treatment modalities have been utilized to treat unicameral bone cyst (UBC), but evidence has not been fully described to support one treatment over another and the optimal treatment is controversial. The aim of this quantitative systematic review was to assess the effectiveness of different UBC treatment modalities.

**Methods:**

We utilized Pubmed to isolate retrospective studies on patients with UBC who received any kind of treatment. The included studies needed to have a minimum sample size of 15 patients, and have provided data on radiographic healing outcome.

**Results:**

Sixty-two articles were selected for the meta-analysis from a total of 463 articles. The cumulative sample size was 3,211 patients with 3,217 UBC, and male to female ratio was 2.2:1. The summary or pool estimate of methylprednisolone acetate (MPA) injection resulted in a healing rate of (77.4 %) that was comparable to bone marrow injection (77.9 %). A higher healing rate was observed with MPA injection when inner wall disruption was performed. The pool estimate of bone marrow with demineralized bone matrix injection was high (98.7 %). UBC healing rate after surgical curettage was comparable whether autograft or allograft was utilized (90 %). UBC treatment with flexible intramedullary nails without curettage provided almost 100% healing rate, while continuous decompression with cannulated screws provided 89 % healing rate. Conservative treatment indicated a healing rate of 64.2, 95 % CI (26.7–101.8).

**Conclusions:**

Active treatment for UBC provided variable healing rates and the outcomes were favorable relative to conservative treatment. Due to the heterogeneity of the studies and reporting bias, the interpretation of these findings should be handled with caution.

## Introduction

Unicameral bone cyst (UBC) is a benign lesion that mostly affects children and adolescents, and represents about 3 % of primary tumors in the first two decades of life [1–3]. UBC is also known as solitary or simple bone cyst and radiographically is seen as mildly expansile, lytic thin-walled bone lesion without periosteal reaction. The typical location is in the metaphysis adjacent to the growth plate of the tubular bones, mostly the humerus and femur [3]. Based on the distance between the cyst and the growth plate, UBC is classified as active when the distance is less than 5 mm, and latent when the distance is larger than 5 mm [4]. Other locations of UBC may include the pelvis, ribs, vertebrae and the tarsal bones, especially calcaneus. Several theories have been postulated to explain the origin of UBC, including trauma and inflammation; however, none has been conclusive [5].

The purpose of treatment is to restore bone strength, cortical thickness and obliteration of the cyst; and based on these criteria, healing of UBC was classified by Neer [3] and Capanna [6]. Complete filling of the cyst with restoration of cortical thickness was described as healed cyst. When small radiolucent areas persist with good bone strength, the cyst was described as partially healed. When the UBC had continuous bone lucency and thin or broken cortex and did not respond to the treatment, the cyst was considered persistent. Cyst recurrence was defined when a cavity and expansile remodelling and thinning of the cortex develop after full obliteration.

Various treatment modalities have been utilized to treat UBC, including surgical and nonsurgical interventions with variable reported healing rates. UBC has frequently been examined in the literature, yet no clearcut evidence supports one modality over another. The aim of this current study was to review the literature and examine the published articles referenced in Pubmed about the healing outcome of UBC management with every treatment modality. A qualitative systematic review was performed to screen all the articles and to select eligible articles for a meta-analysis on UBC treatment outcome. We postulated that treatment of UBC leads to better rates of cyst healing.

## Materials and methods

### Search techniques and terms:

**Fig. 1:**
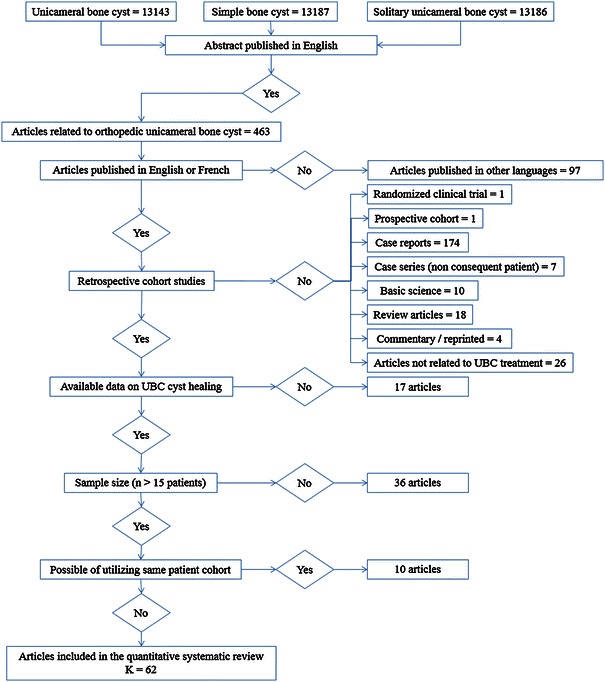
Schema illustrating the details of the articles for the systematic review and the meta-analysis

The studies were identified from Pubmed until 2012 using the following search terms: unicameral bone cyst, simple bone cyst and solitary bone cyst. The articles were searched by a single investigator (MK), but were reviewed by two investigators (MK, AK) independently and data were extracted during the review. The articles selected were reviewed and screened for pertinent information based on specific criteria (Fig. 1).

### Eligibility criteria

The extracted articles were examined based on: (a) non-experimental retrospective studies on unicameral bone cyst in any anatomical location and any kind of treatment, (b) studies conducted in any geographic location, (c) studies with abstracts published in English, (d) studies that examined consecutive patients with sample size equal or greater than 15 patients, (e) studies with follow up more than 6 months, and (f) studies that demonstrated the radiographic outcome of treatment that can be classified into (healed, partially healed, persistence or recurrence).

Studies with sample size smaller than 15 were excluded in order to avoid sparse data bias; implying negative finding as a result of small sample size and limited statistical power. Case reports were excluded, as they did not examine consecutive patients. When more than one study was conducted in the same institution, the articles were examined for a possibility of duplicate report on the same patient sample, and data were retrieved from the most complete or recent study.

### Study quality assessment

The included studies were assessed for study design, sampling techniques and data on patients’ characteristics and radiographic healing status. We determined the objectivity of each study, whether the aims or the purposes were clearly stated, and whether the studies presented factual results. We also examined for adequate statistical analysis and how the outcomes were determined. Studies were assessed for confounding factors that might have influenced the outcome and for any bias, including selection, information and misclassification biases. This quality assessment provided comparable measures to the preferred reporting for systematic reviews and meta-analysis (PRISMA statement) [7].

### Study variables

Each study was identified by the first author’s last name and the year of publication. Study populations were stratified by sex, age at treatment, cyst location, cyst activity (active or latent), follow-up time and radiographic healing classified by Neer [3] and Capanna [6] criteria. The cohort of each study was categorized into two groups based on the final healing status of UBC: success—good outcome included healed and partially healed UBCs, and failure—poor outcome included persistence and recurrent UBCs.

### Treatment groups

All treatment modalities were included in this study (conservative and surgical intervention). Studies that utilized injection as a method of treatment were categorized in one treatment group and subgroups were identified based on the material utilized for injection, including; methylprednisolone acetate (MPA) injection, bone marrow injection, combined MPA with bone marrow, combined bone marrow with demineralized bone matrix (DBM), and combined bone marrow with MPA and DBM. The technique of injection procedure was reviewed in each article to identify whether inner wall disruption was performed or not, and based on this criterion, every injection subgroup was subcategorized into injection with and without inner wall disruption. Studies on surgical treatment were examined and subgroups were identified, including: curettage only, curettage with bone graft (autograft versus allograft), curettage with bone substitution (calcium sulphate pellets, calcium phosphate pellets, Hydroxyapetite, ceramic substitute), curettage with myoplasty, cyst excision, flexible intramedullary nailing (IM nail) and continuous decompression with cannulated screws.

### Comparative studies

Retrospective comparative studies that presented outcome of different treatment modalities were selected for further evaluation by combining the patients of each treatment group. Comparative assessment performed included: MPA vs. bone marrow injection, MPA injection with vs. without inner wall disruption, curettage with autograft vs. curettage with allograft, MPA injection vs. curettage with autograft, MPA injection vs. curettage with allograft.

### Anatomical location

We identified the studies that provided a list of UBC cases demonstrating age, sex, cyst location treatment modality and radiographic healing outcome. The UBC cases were categorized based on the anatomic location for further assessment.

### Sample size and power estimation

Four hundred and sixty-three articles published between 1942 and 2012 were identified for the qualitative systematic review. The number of studies included in the quantitative systematic review (meta-analysis or quantitative evidence synthesis) was represented by *K* = 62. The combination of study sizes (UBC) that constituted this meta-analysis was represented by *n* = 3,217 cysts. To estimate the power of this meta-analysis, we used 95 % confidence interval (5 % error), effect size of 20 % (0.2), and a random effect meta-analytic technique (DerSimonian and Laird). With these parameters, and with the combined sample sizes from the individual studies (*n*) for each treatment group, we obtained sufficient power (>80 %) to detect a difference between success (good) and failure (poor) outcomes in terms of UBC healing.

### Statistical analysis

Frequency and percentages were employed to summarize nominal variables, while mean and standard deviation were used to describe the continuous variables, including age and follow-up.

We tested the hypotheses to determine the effectiveness of different treatment modalities. Both the fixed method of Peto and Mantel–Haenszel, and the random effect method of Dersimonian-Laird meta-analytic techniques were utilized. The fixed effect method was applicable to the summary point estimate when the studies that constituted the meta-analysis were assessed to be homogenous (implying heterogeneity test I with a significance level >0.05). Unless the homogeneity test was performed, the fixed method was inapplicable. The random effect method was utilized when the homogeneity assumption was not met.

The significance level was <0.05 and all tests were two-tailed. All analyses were performed using STATA 12.0 (StataCorp LP, College Station, TX).

## Results

The results of this meta-analysis consisted of two parts, descriptive and inferential. Four hundred and sixty-three articles were identified for the qualitative systematic review. Sixty-two articles were eligible for the quantitative systematic review [3, 6, 8–67]. The rest of the articles were excluded, including: 97 articles that were not in English or French [68–164], one randomized clinical trial [165], one prospective study [166], seven case series [167–173], 174 case reports [2, 174–346], 10 basic science articles [5, 347–355], 26 articles that did not discuss UBC treatment [1, 356–380], 17 articles that did not represent clear data on healing [381–397], 18 review articles [4, 398–414], one article that was a reprint [415], three commentary articles [416–418], 36 articles with a sample size smaller than 15 [419–454], and ten articles with possibility of shared cohort [455–464] (Fig. 1).

The 62 studies of the meta-analysis generated 3,211 patients with 3,217 UBCs. There were 1,709 boys and 765 girls. While some studies did not provide enough data on gender and the proportions did not add up to 100 %, the estimated male to female ratio was 2.2:1. The clinical presentation was with a pathologic fracture in 1,107 UBCs, pain in 204 cysts, and incidental finding in 133 UBCs (the clinical presentation was not specified in all studies). Seven hundred and twenty-four UBC were classified as active and 535 cysts were latent. The overall average age at treatment was 10.9 ± 2.9 years, and the average follow up was 6.1 ± 4.2 years (not all the studies provided data on age at surgery and follow-up).

The most common location of UBC was in the humerus (59.2 %), followed by the femur (25.9 %). The calcaneus was affected in 6 %, the tibia in 4 %, the fibula in 1.5 %, the radius in 1.1 %, the pelvis in 0.9 %, and the other locations were affected in 1.4 % (Table 1).

**Table 1:** Number of UBC cases stratified by the anatomic location

Anatomic location	Number of UBC
Humerus^a^	1,629
Proximal humerus	691
Mid humerus	123
Distal humerus	13
Radius	29
Ulna	7
Scapula	1
Clavicle	3
Pelvis^a^	26
Ilium	14
Ischium	3
Pubis	5
Femur^a^	712
Proximal femur	303
Mid femur	22
Distal femur	27
Tibia^a^	111
Proximal tibia	19
Distal tibia	24
Fibula^a^	40
Proximal fibula	9
Mid fibula	3
Distal fibula	3
Calcaneus	166
Talus	3
Metatarsus	6
Other locations	20

^a^The exact location of the cyst was not determined in all the papers; therefore, the count did not add up to 100 %

Based on radiographic healing classification, 2,448 UBCs (76.1 %) were classified as healed or partially healed (success), while 769 cysts (23.9 %) were classified as persistent or recurrent (failure).

### Treatment

Primarily three treatment modalities were assessed; injection, surgery and conservative treatment (observational management) (Table 2). Overall injection was utilized in 1,370 cysts. Of the injection cases, 1,128 cysts were treated with MPA, while 114 cysts were injected with bone marrow. Surgical treatment included several surgical techniques as presented in Table 2. Curettage as a separate treatment was done in 31 UBCs. Curettage with allograft was performed in 353 UBCs, while 128 cysts were treated with curettage and autograft. Flexible IM nail was used in 205 UBCs and cannulated screws for continuous decompression were utilized in 61 UBCs. Conservative treatment implying observational management was utilized in 149 cysts.

**Table 2:** Outcome of UBC stratified by the treatment modalities

Treatment modality	*n* = UBC	Treatment outcome		Reference articles
		Success	Failure	
Injection (MPA)	1,128	806	322	[6, 12, 14, 16, 19, 22, 25–29, 33, 37–39, 41, 44, 47, 50, 55, 64, 66]
Injection (bone marrow)	114	88	26	[19, 26, 38–40, 45]
Injection (bone marrow + DBM)	85	73	12	[15, 21, 33, 45]
Injection (MPA + bone marrow)	9	3	6	[23]
Injection (bone marrow + MPA + DBM)	34	17	17	[37]
Only curettage	31	16	15	[9, 18, 38, 43, 46, 47]
Curettage with allograft	353	271	82	[8, 9, 14, 25, 27, 29, 35, 37, 53, 57–60]
Curettage with allograft + bone marrow	23	21	2	[56]
Curettage with autograft	128	96	32	[9, 14, 19, 25, 27, 36, 43, 46, 47, 58, 59]
Curettage with allograft + autograft	3	3	0	[14, 59]
Curettage with pellets	115	104	11	[19, 23, 24, 42, 47, 48, 59, 63]
Curettage with hydroxyapetite	25	22	3	[47, 48]
Curettage with ceramic	12	12	0	[8]
Curettage + Zinc cauterization of the cyst	9	8	1	[43]
Curettage with osteoset pellets + autograft	1	1	0	[59]
Curettage with myoplasty	35	32	3	[10]
Curettage with bone graft + Kuntcher stabilization (rinsing the cyst cavity with peroxide)	12	9	3	[64]
Curettage with bone graft + plate stabilization	10	9	1	[66]
Curettage with pellets + plate stabilization	7	6	1	[23]
Minimal invasive curettage + ethanol cauterization + calcium sulfate pellet + cannulated screw	12	11	1	[23]
Curettage + autograft + allograft with IM nail stabilization	19	11	8	[51]
Papers with different surgical techniques^a^	528	426	102	[3, 12, 13, 17, 18, 28, 34, 52, 62, 64]
Cyst excision	83	76	7	[9, 14, 36, 54, 65, 66]
Cyst wall trepanation	26	26	0	[9, 49]
IM nail without curettage	205	196	9	[11, 16, 19, 20, 30–32, 67]
Continuous decompression with cannulated screws	61	47	14	[13, 29, 61]
Conservative treatment	149	58	91	[9, 12, 13, 36, 44, 47, 52, 58–60, 66]
Overall number of cysts	3,217	2,448	769	

^a^These papers were not included in the subcategories for treatment modalities, because healing classification was not stratified based on the treatment

### Comparative studies

**Fig. 2:**
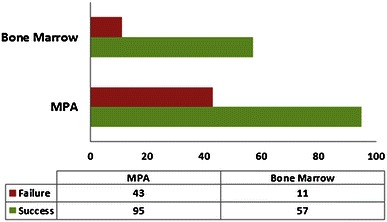
*Bar chart* illustrating the studies that compared the difference in healing outcome between bone marrow injection and methylprednisolone (MPA) injection

**Fig. 3:**
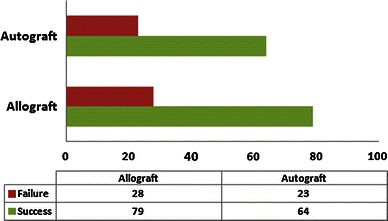
*Bar chart* illustrating the studies that compared the difference in healing outcome of surgical curettage between allograft and autograft

**Fig. 4:**
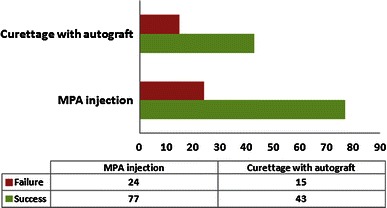
*Bar chart* illustrating the studies that compared the difference in healing outcome between methylprednisolone (MPA) injection and surgical curettage with autgraft

**Fig. 5:**
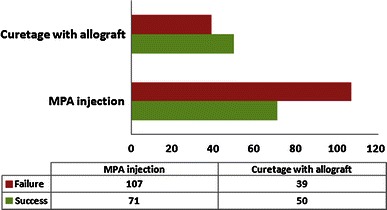
*Bar chart* illustrating the studies that compared the difference in healing outcome between methylprednisolone (MPA) injection and surgical curettage with allograft

We examined the studies that compared the healing outcome of MPA injection with bone marrow injection [19, 26, 38, 39]. Of 206 patients in this protocol, a higher rate of success was observed in bone marrow injection compared to MPA injection (Fig. 2). Only one study examined MPA injection outcome with and without inner wall disruption [47] and the outcome success was comparable. Five studies examined the difference in outcome of curettage with allograft compared to autograft [3, 9, 14, 25, 27], and the success rate was comparable (Fig. 3). Five articles compared the outcome of MPA injection relative to surgical curettage with autograft [14, 19, 25, 27, 47] (Fig. 4), and five articles also examined the outcome of MPA injection compared to surgical curettage with allograft [14, 25, 27, 29, 37] (Fig. 5).

### Outcome of treatment based on the anatomic location

The outcome of treatment based on UBC anatomic location was also examined, including (humerus, femur, calcaneus, fibula, tibia and radius). Some studies did not present healing status for each anatomic location of the cyst; therefore, the numbers and percentages did not add up to 100 % of the total number of UBC of the meta-analysis. Tables 3, 4, 5, and 6 present the findings on healing outcome stratified by sex, activity level and treatment modalities. A few studies examined the fibula, radius, tibia and pelvic locations.

**Table 3:** The characteristics and healing outcome of UBCs located in the humerus (*n* = 497)

	Treatment outcome	
	Success	Failure
Sex^a^		
Male	132 (79.0)	35 (21.0)
Female	51 (82.3)	11 (17.7)
Location^a^		
Metaphysis	145 (73.6)	52 (26.4)
Diaphysis	31 (93.9)	2 (6.1)
Activity^a^		
Active	31 (72.1)	12 (27.9)
Latent	31 (81.6)	7 (18.4)
Treatment		
Surgical curettage	107 (77.0)	32 (23.0)
No graft	12 (63.2)	7 (36.8)
Allograft	23 (95.8)	1 (4.2)
Autograft	35 (74.5)	12 (25.5)
Mix allograft + autograft	11 (57.9)	8 (42.1)
Allograft + bone marrow	7 (78.5)	1 (12.5)
Ceramic	6 (100)	0 (0)
Osteoset pellets	13 (81.2)	3 (18.8)
Injection		
MPA	86 (81.1)	20 (18.9)
Bone marrow	16 (72.7)	6 (27.3)
Bone marrow + DBM	28 (100)	0 (0)
MPA + bone marrow	2 (100)	0 (0)
IM nail	88 (96.7)	3 (3.3)
Screws	12 (85.7)	2 (7.1)
Cyst excision	39 (92.9)	3 (14.7)
Conservative	12 (22.6)	41 (77.3)
Proximal metaphysis	6 (12.8)	41 (78.2)
Diaphysis	4 (100)	0 (0)

Data were retrieved from these references: [6, 8, 9, 12, 13, 15, 21, 24, 25, 27, 30, 31, 38, 42, 43, 45, 46, 51, 54, 61, 65, 67]

*MPA* methylprednisolone, *DBM* demineralized bone matrix, *IM nail* Intramedulary nail, *Screws* continuous decompression with cannulated screws

^a^The exact location of the cyst was not determined in all the papers; therefore, the percentages did not add up to 100 %

**Table 4:** The characteristics and healing outcome of UBCs located in the femur (*n* = 211)

	Treatment Outcome	
	Success	Failure
Sex^a^		
Male	37 (75.5)	12 (24.5)
Female	30 (88.2)	4 (11.8)
Location^a^		
Metaphysis	99 (84.6)	18 (15.4)
Diaphysis	6 (100.0)	0 (0)
Activity^a^		
Active	30 (75.0)	10 (25.0)
Latent	16 (84.2)	3 (15.8)
Treatment		
Surgical curettage	63 (81,1)	14 (18.2)
No graft	13 (76.5)	4 (23.5)
Allograft	15 (88.2)	2 (11.8)
Autograft	18 (78.3)	5 (21.7)
Ceramic	7 (100)	0 (0)
Osteoset pellets	6 (85.7)	1 (14.3)
Injection		
MPA	46 (76.7)	14 (23.3)
Bone marrow	10 (76.9)	3 (23.1)
Bone marrow + DBM	10 (100)	0 (0)
IM nail	32 (97.0)	1 (3.0)
Screws	2 (40.0)	3 (60.0)
Cyst excision	2 (100)	0 (0)
Conservative	7 (63.3)	4 (36.4)
Metaphysis	4 (50.0)	4 (50.0)

Data were retrieved from these references: [6, 8, 9, 12, 13, 15, 20, 21, 24, 25, 27, 30, 31, 38, 42–46, 54, 61]

*MPA* methylprednisolone, *DBM* demineralized bone matrix, *IM nail* Intramedulary nail, *Screws* continuous decompression with cannulated screws

^a^The exact location of the cyst was not determined in all the papers; therefore, the percentages did not add up to 100 %

**Table 5:** The characteristics and healing outcome of UBCs located in the calcaneus (*n* = 116)

	Treatment outcome	
	Success	Failure
Sex^a^		
Male	24 (77.4)	7 (22.6)
Female	19 (95.0)	1 (5.0)
Treatment		
Surgical curettage	76 (96.2)	3 (3.8)
No graft	1 (100)	0 (0)
Allograft	33 (100)	0 (0)
Autograft	17 (94.4)	1 (5.6)
Mix allograft + autograft	2 (100)	0 (0)
Allograft + bone marrow	21 (91.3)	2 (8.7)
Osteoset pellets	2 (100)	0 (0)
Injection (bone marrow + DBM)	1 (100)	0 (0)
Screws	10 (100)	0 (0)
Cyst excision	1 (100)	0 (0)
Conservative	1 (4.0)	24 (96.0)

Data were retrieved from these references: [21, 24, 42, 54, 56–59, 61]

*DBM* demineralized bone matrix, *Screws* Continuous decompression with cannulated screws

^a^The exact location of the cyst was not determined in all the papers, therefore the percentages did not add up to 100 %

**Table 6:** The characteristics and healing outcome of UBCs in other locations

Radius (*n* = 8^a^)		
Surgical curettage	6 (100)	0 (0)
Injection		
Bone marrow + DBM	1 (100)	0 (0)
Cyst excision	1 (100)	0 (0)
Fibula (*n* = 5^b^)		
Injection		
MPA injection	2 (100)	0 (0)
Bone marrow + DBM	1 (100)	0 (0)
Cyst excision	2 (100)	0 (0)
Tibia (*n* = 20)^c^		
Surgical curettage injection	9 (90)	1 (10)
MPA	3 (60)	2 (40)
Bone marrow	2 (100)	0 (0)
IM nail	1 (100)	0 (0)
Screws	1 (100)	0 (0)
Ischium (*n* = 3)^d^		
Surgical curettage	1 (100)	0 (0)
Cyst excision	1 (100)	0 (0)
Screws	1 (100)	0 (0)
Ilium (*n* = 3)^e^		
Curettage	1 (50)	1 (50)
Screws	0 (0)	1 (100)
Pubis (*n* = 1)^f^		
Screw	1 (100)	0 (0)
Talus (*n* = 1)^g^		
Injection (bone marrow + DBM)	1 (100)	0 (0)
Clavicle (*n* = 1)^h^		
Cyst excision	1 (100)	0 (0)

*MPA* methylprednisolone, *DBM* demineralized bone matrix, *IM nail* intramedulary nail, *Screws* continuous decompression with cannulated screws

^a^The exact location of UBCs in the radius was not specified, reference: [12, 21, 24, 38, 46, 54]

^b^Among the UBCs in the fibula, three cysts were in the metaphysic, two were not specified, reference: [6, 9, 21, 27]

^c^Among the UBCs in the tibia, 14 cysts were in the metaphysis, two were in the diaphysis and the rest were not specified, reference: [6, 8, 9, 20, 25, 27, 43, 45, 46, 61]

^d^Ref. [9, 54, 61]

^e^Ref. [9, 43, 61]

^f^Ref. [61]

^g^Ref. [21]

^h^Ref. [9]

### Quantitative results (Pool estimates)

**Fig. 6:**
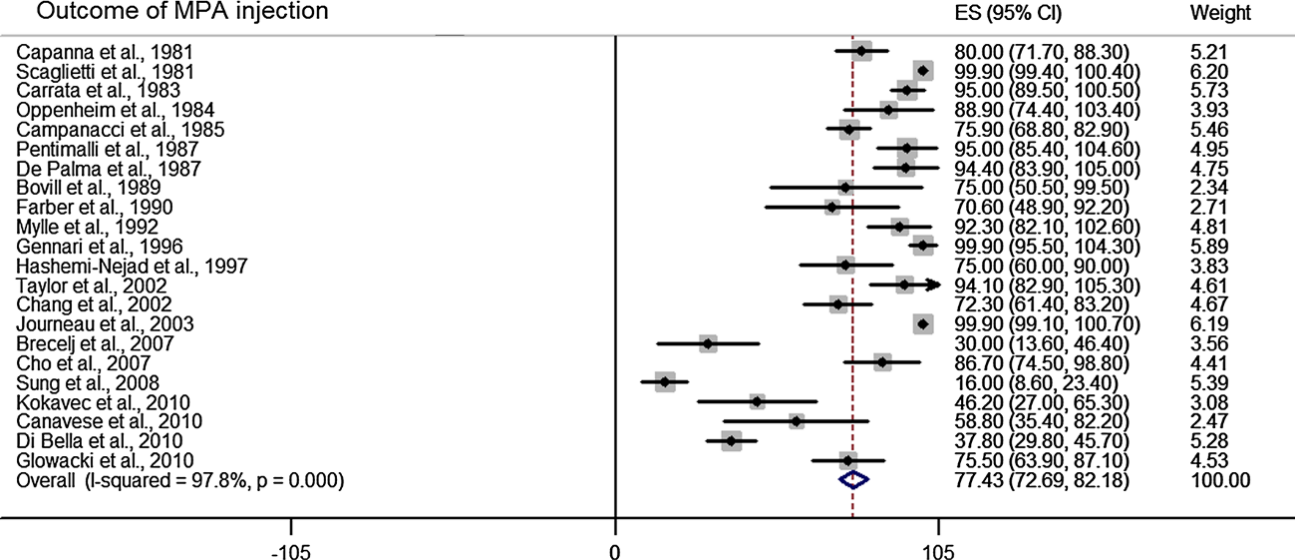
*Forest plot* illustrating the point estimate of each article and the pool estimate of healing outcome of methylprednisolone (MPA) injection

**Fig. 7:**
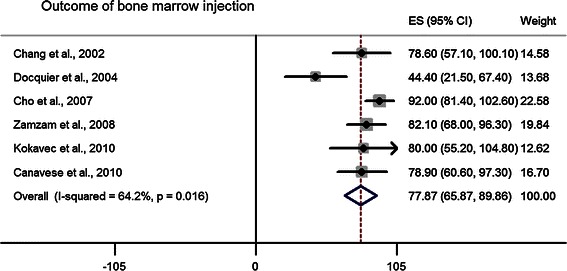
*Forest plot* illustrating the point estimate of each article and the pool estimate of healing outcome of bone marrow injection

Table 7 illustrates the percentages and 95 % CI of each treatment modality representing the successful outcome (healed and partially healed UBC). The combination of studies in almost all the categories of the meta-analysis indicated satisfactory healing of UBC after each specific treatment. The summary or pool estimate of overall injection category was 81.3, 95 % CI (77.9–84.8), implying a statistically significant 81 % success rate for overall injection (*p* < 0.05). MPA injection resulted in a healing rate of 77.4, 95 % CI (72.7–82.2) (Fig. 6). The effect of inner wall disruption with MPA injection was examined and higher healing rate was observed when the disruption was performed. The pool estimate of bone marrow injection was comparable to MPA injection, 77.9, 95 % CI (65.9–89.9) (Fig. 7). Only one study reported on the outcome of bone marrow injection with inner wall disruption and success rate was 78.6 % [39]; therefore, no pool estimate was performed for bone marrow injection with inner wall disruption. The pool estimate of bone marrow with DBM injection was high, 98.7, 95 % CI (95.7–101.7) relative to injection with either MPA or bone marrow.

**Table 7:** Pool estimate of treatment outcome for different treatment modalities

Treatment category	Pool estimate (%)	95 % Confidence interval
Overall injection^a^	81.3	77.9–84.8
MPA injection^a^	77.4	72.7–82.2
With no inner wall disruption^a^	77.5	72.7–82.3
With inner wall disruption^a^	86.6	59.6–113.7
Bone marrow injection^a^	77.9	65.9–89.9
With no inner wall disruption^a^	77.4	63.2–91.5
Bone marrow + DBM injection^a^	98.7	95.7–101.7
With no inner wall disruption^b^	99.9	98.7–101.1
With inner wall disruption^a^	86.1	57.6–114.7
Curettage with allograft^a^	90.8	86.7–94.9
Curettage with autograft^a^	90.9	85.8–96.1
Curettage with pellets^a^	96.5	91.7–101.3
Sulfate pellets^a^	89.9	75.3–104.4
Phosphate pellets^b^	99.2	95.7–102.7
Curettage with hydroxyapetite^b^	98.7	94.5–102.9
Cyst excision^a^	96.7	91.3–102.1
IM nail with no curettage^b^	99.7	99.0–100.4
Continuous decompression with cannulated screws^a^	83.9	63.1–104.7
With no curettage^a^	88.8	64.5–113.0
Cyst wall trepanation^b^	99.9	98.7–101.1
Conservative treatment^a^	64.2	26.7–101.8

^a^Pool estimate was reported utilizing random effect meta-analysis

^b^Pool estimate was reported utilizing fixed effect meta-analysis

**Fig. 8:**
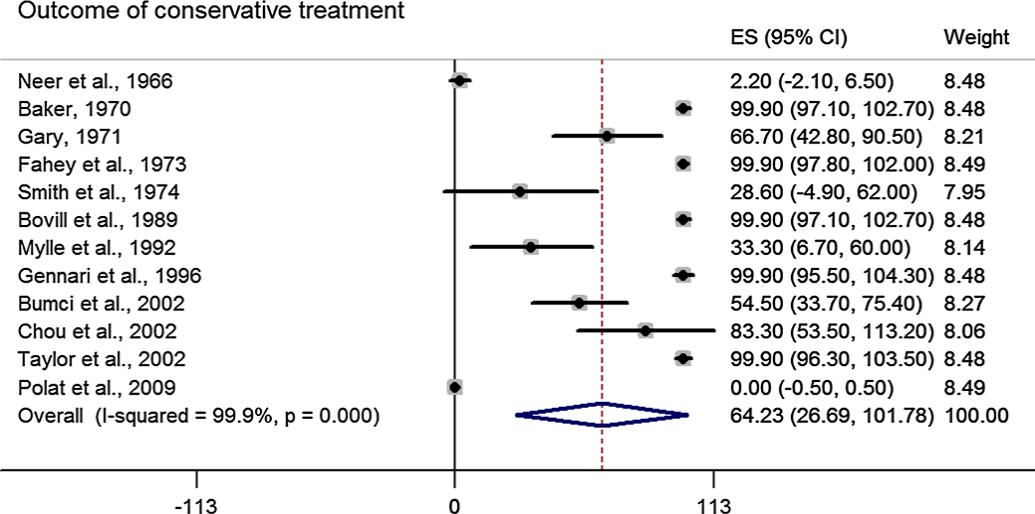
*Forest plot* illustrating the point estimate of each article and the pool estimate of healing outcome of conservative treatment

Surgical curettage was examined according to the utilization of bone graft or bone substitute, and the pool estimates of all the subcategories were comparable. Other surgical managements were also assessed, including cyst excision, continuous decompression with IM nail or cannulated screws, and cyst wall trepanation (Table 3). The pool estimate of conservative treatment was 64.2, 95 % CI (26.7–101.8) (Fig. 8).

## Discussion

The effect of various treatment modalities on UBC management has been repeatedly examined in the literature, but the natural history of UBC and the optimal treatment remain unclear. The conventional knowledge about UBC is that it is a self-limited benign bone lesion [370]; however, the healing rate without treatment (observational management) is undetermined and most of the studies in this review demonstrated better results with active treatment. With the purpose to better understand the role of each treatment modality of UBC, we aimed in this quantitative systematic review to synthesize the literature and assess the natural history of UBC, as well as evaluate the various treatment methods.

The main purpose of meta-analysis is to improve the power by combining homogeneous studies and arriving at a summary effect or pool estimate. We performed both systematic review and meta-analysis to examine the effect of several treatment modalities on UBC. Specifically, we tested the hypothesis regarding the effectiveness of different treatment modalities on UBC healing. Our meta-analysis has some relevant findings: overall active treatment improves UBC healing, regardless of anatomic site and the type of treatment compared to observational management. In this meta-analysis, observational treatment leads to a healing rate of 64 %. However, due to the variability of the studies, the rate of healing after conservative treatment can be as low as 27 % and as high as 100 %. The healing rate after conservative treatment varied in different anatomic location. Higher failure rate of conservative treatment was found in calcaneal and humeral UBCs compared to femoral UBCs (although the number of femoral UBCs was small, 11 out of 211 cysts were treated conservatively).

The indication to treat UBC is to prevent a pathologic fracture and to manage symptoms, especially pain. Historically, surgical curettage and cyst excision with bone graft were the optimal choices to treat UBCs. Based on this meta-analysis, UBC healing rate after surgical curettage was comparable (90 %) whether autograft or allograft was utilized. The utilization of MPA in the treatment of UBC was first described by Scaglietti [454]. The role of MPA is to reduce the production of cyst fluid from the inner wall cyst, which enhances bone healing [352]. On the other hand, bone marrow injection was proposed to accelerate healing, given the osteogenic potential of red marrow [465, 466]. In this meta-analysis, the healing rate of UBC was comparable after injection with bone marrow or MPA (77 %). The healing rate after MPA or bone marrow injection was higher (almost double) in our meta-analysis compared to the results demonstrated by Wright et al. [165] in a randomized (prospective) clinical trial. This might be a result of failure to report unsuccessful outcomes in studies of retrospective design (reporting bias). Both the randomized study by Wright et al. [165] and our meta-analysis demonstrated comparable healing rate after MPA injection and bone marrow injection.

The utilization of IM nails or cannulated screws for the treatment of UBC was also described, and the rationale was that theoretically, UBCs develop from venous blockage and increase of fluid pressure inside the cyst [5]. In this meta-analysis, we found that treatment with IM nails without curettage provided almost 100 % healing rate of UBC in long bones. IM nails were described as an efficient acute treatment for UBCs that present with a fracture [11, 16, 67]. Cyst wall drilling and trepanation also provided a healing rate of almost 100 %; however, this healing rate was estimated based on only two studies [9, 49].

Although this meta-analysis may be interpreted as a comparison between different treatment modalities such as MPA and bone marrow injection, this is incorrect. Studies that tested hypothesis with respect of comparative effectiveness of treatment were not available; therefore, we were unable to use formal meta-analysis method to examine the differences in outcomes of treatment with different treatment modalities. Future studies designed to compare different modalities of treatment are recommended, and will provide the opportunity for these questions to be addressed.

Our systematic review is limited in the sense that different studies presented with different sampling designs and different study protocols. For example, data were not available on MPA dosage that was utilized for treatment. Also, the variability of surgical techniques and type of bone graft (autograft or allograft) has a tendency of introducing a confounding that may lead to bias in our outcomes estimation and interpretation. In addition, we were unable to examine the need for further intervention and recurrence rate in each treatment modality, as data were not clearly specified in the reviewed articles. Also UBC activity as described by the distance from the growth plate may be an important factor that may affect on cyst healing after different treatment modalities. Some of the studies provided data on cyst activity, but only a few of these provided data on the outcome of treatment stratified by cyst activity. The other limitations are the heterogeneity of the patients in terms of UBC anatomic locations, cyst size, sex variability, and duration of treatment and follow-up. We could have performed subgroup meta-analysis in terms of the impact of sex or anatomic site of the UBC on the outcome implying cyst healing. However, since many studies did not provide relevant data, performing such subgroup analysis could have ended up in biased results regarding such effects. Considering this inability to perform the observed subgroup analyses, we recommend future studies to provide complete data on patients’ sex and specific anatomic location.

Heterogeneity remains an important factor to be considered in the conduct and interpretation of meta-analysis. There is no meta-analytic study without some influence of heterogeneity. Several studies used in our meta-analysis showed significant heterogeneity; however, our meta-analysis is not completely driven by this variability. A meta-analytic finding could be reliable and valid despite heterogeneity when appropriate analysis is used in the summary estimate. We applied the random effect meta-analysis given the heterogeneity of studies included in this meta-analysis. Consequently, by adjusting for the differences between studies with the random effect method approach, we have produced relatively standard and valid meta-analytic results on the effect of treatments on the outcomes of UBC. Whereas qualitative systematic review is not intended to quantify results of reviewed literature, we attempted to summarize proportions with regard to UBC healing, and caution is recommended in the interpretation of the results of our systematic review.

We are unaware of previous meta-analysis in this direction to either support or negate our findings. Clinicians will be interested in assessing the possibility of the results of this meta-analysis in developing guidelines regarding the treatment of UBC. Our meta-analysis of published literature on UBC treatment indicates improved healing rates among treated patients relative to the observational management. Healing rate was found to be comparable in studies that utilized bone marrow injection or MPA, and higher rate of healing was found when DBM was added. Surgical curettage resulted in healing rate of 90 % with the utilization of autograft, allograft or any bone substitution material. Healing rate was also high with the utilization of IM nails.
